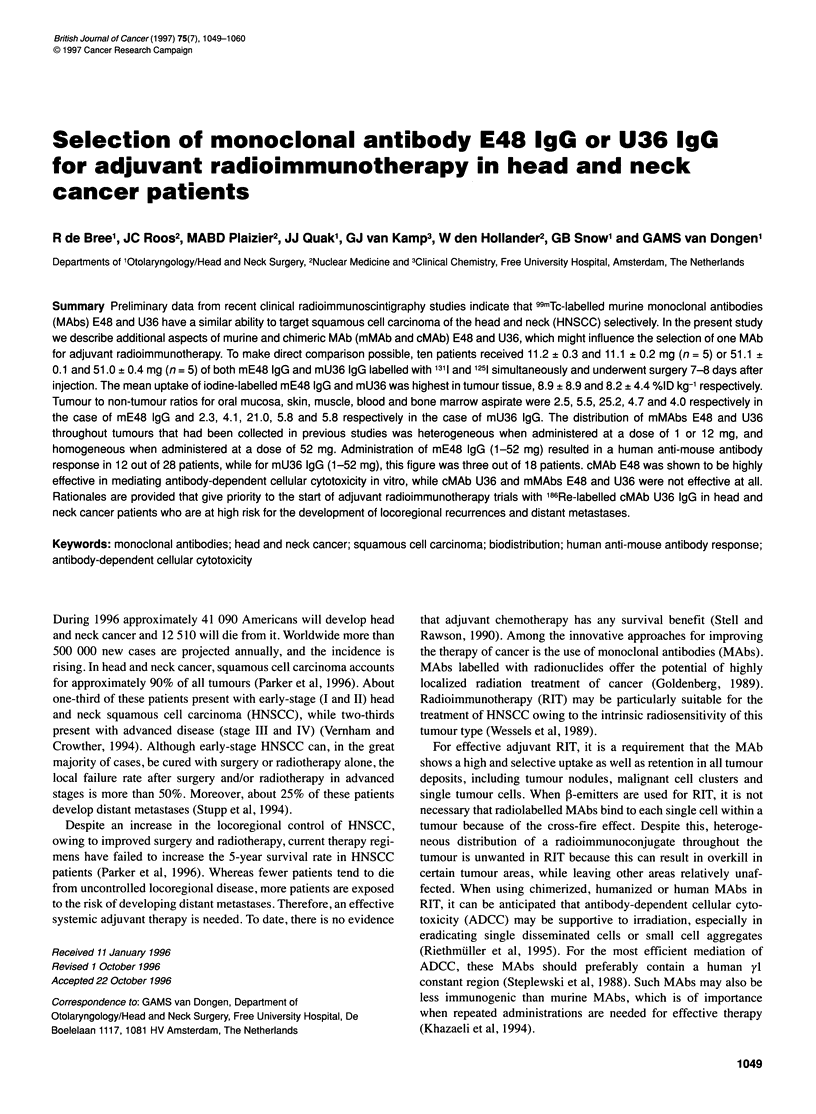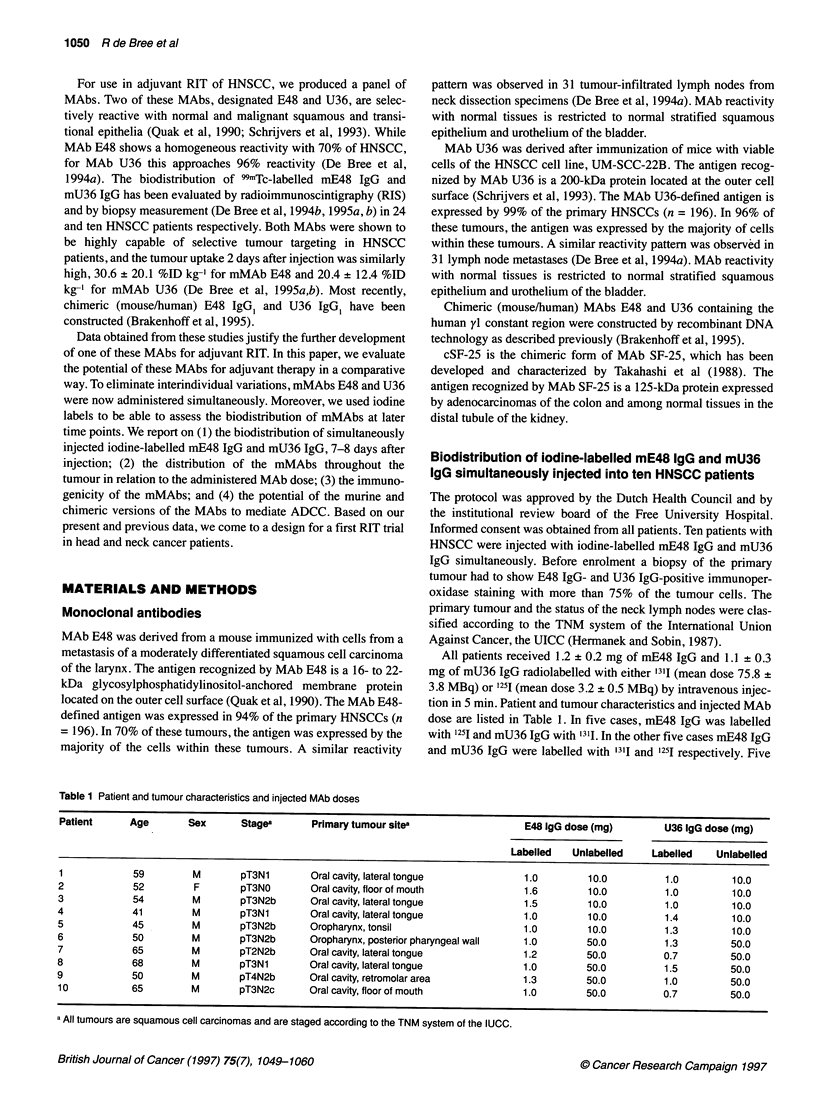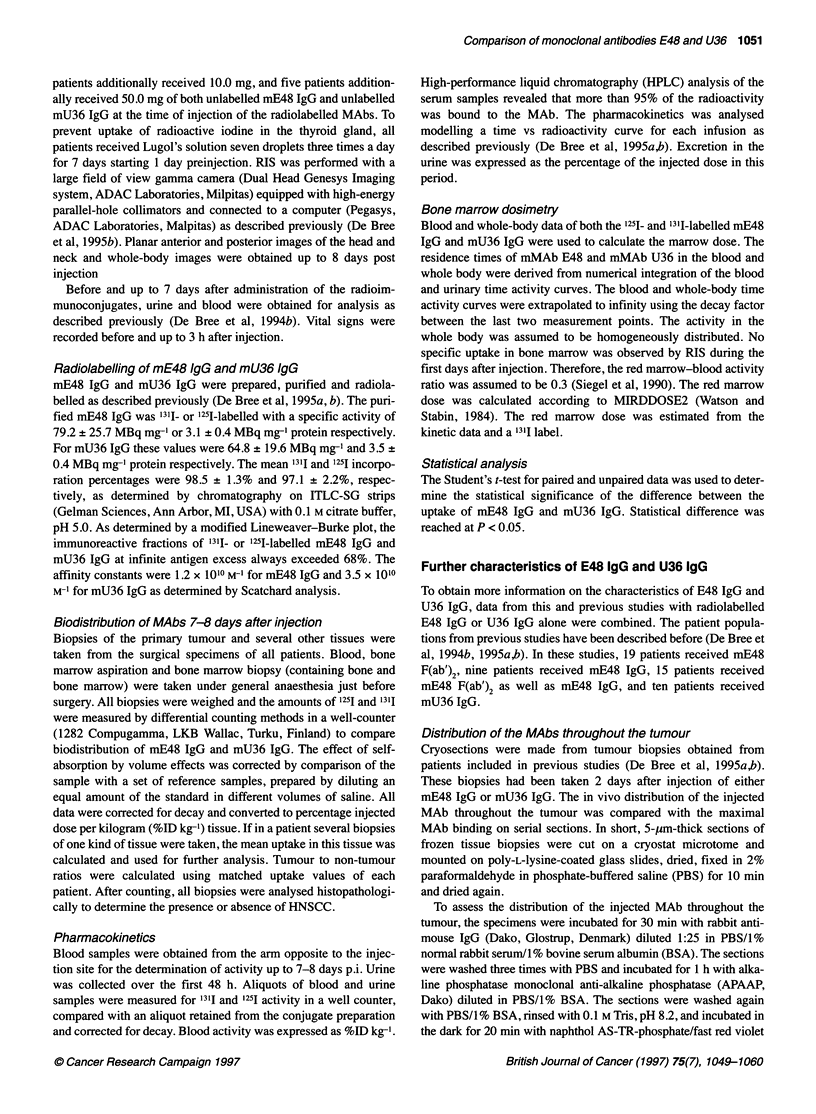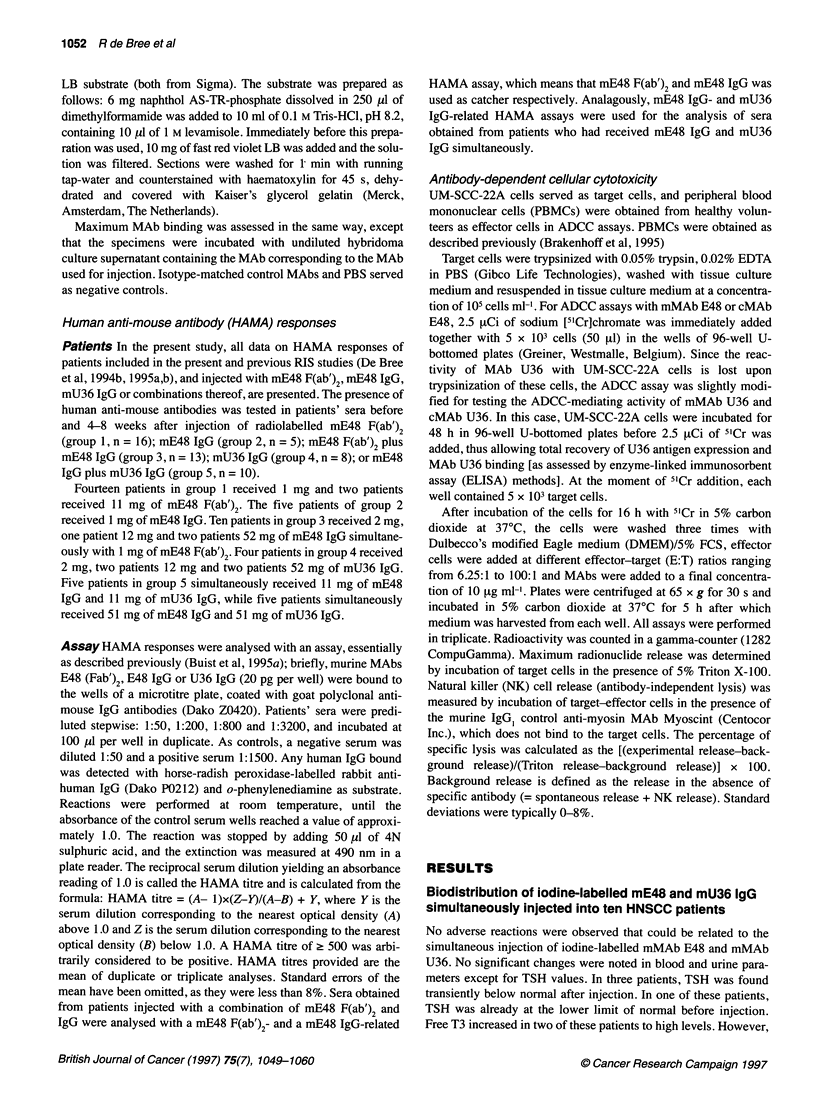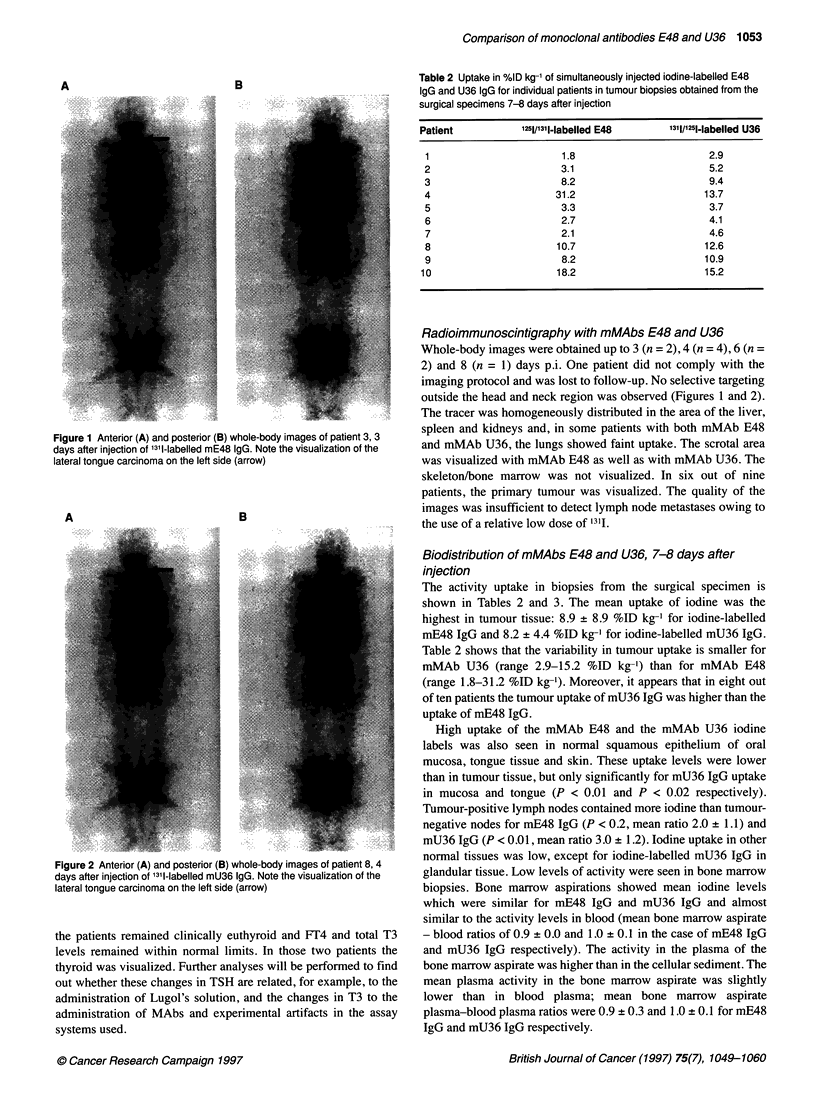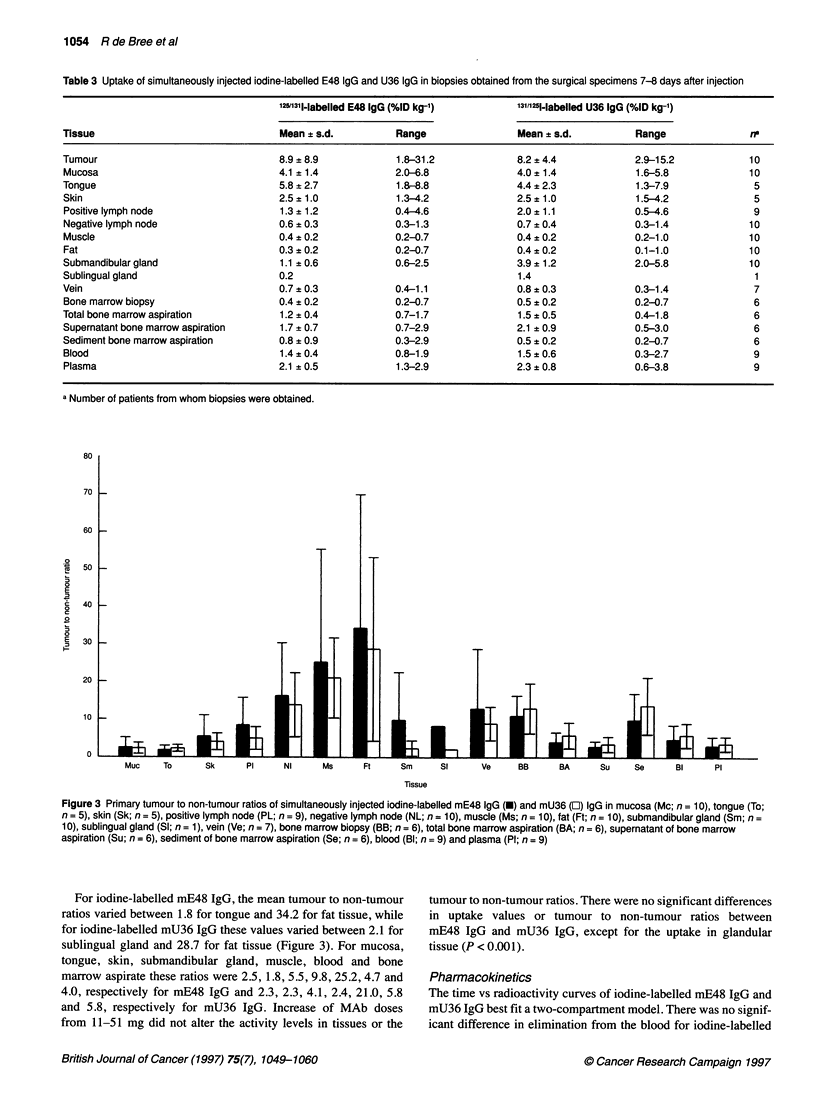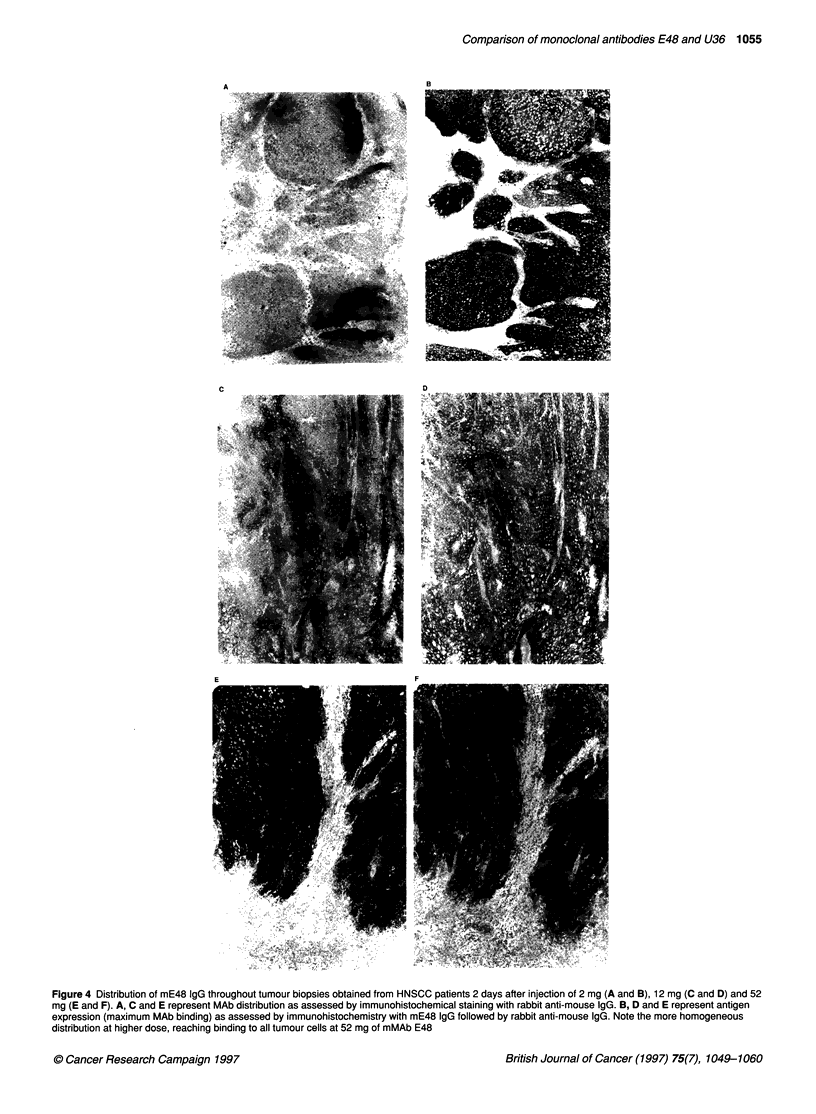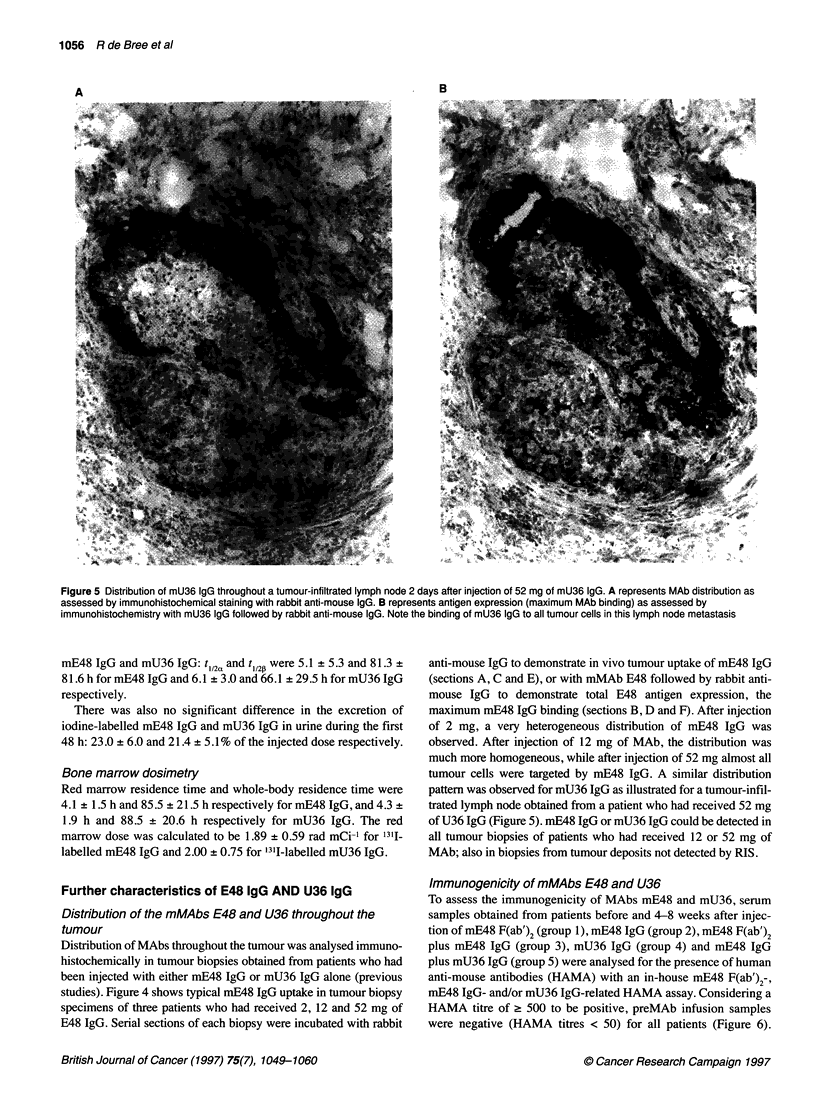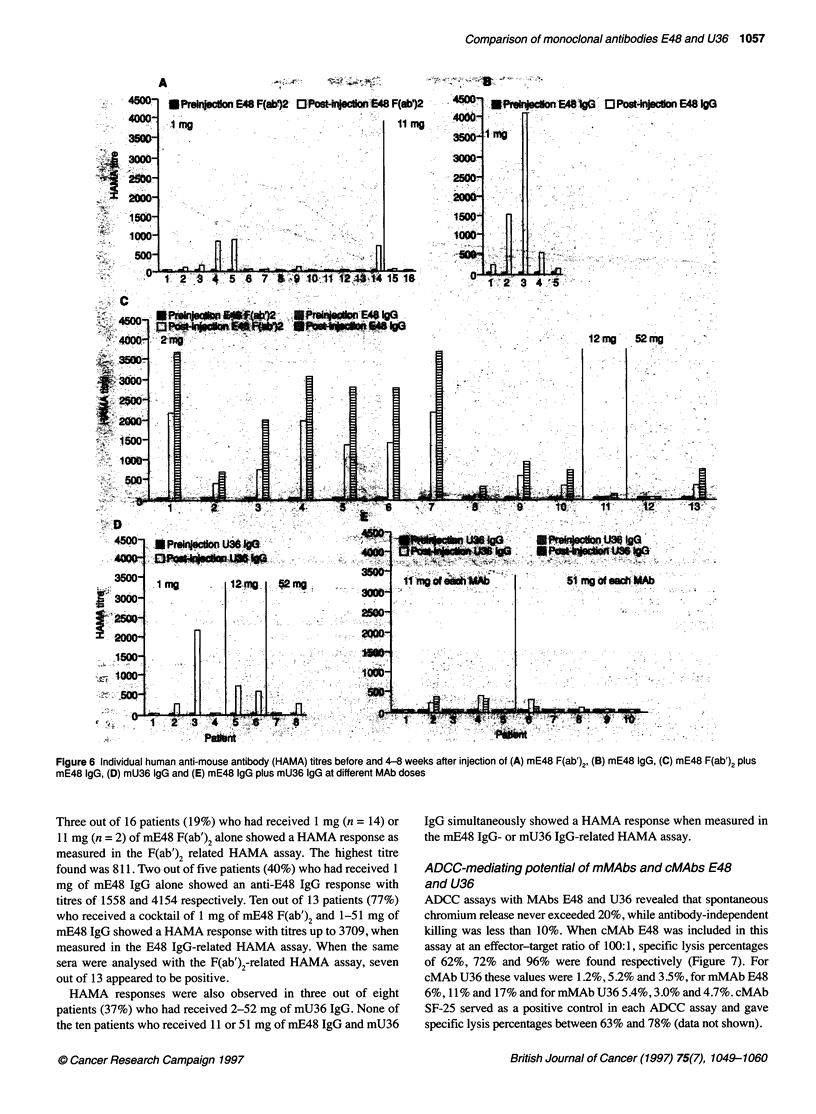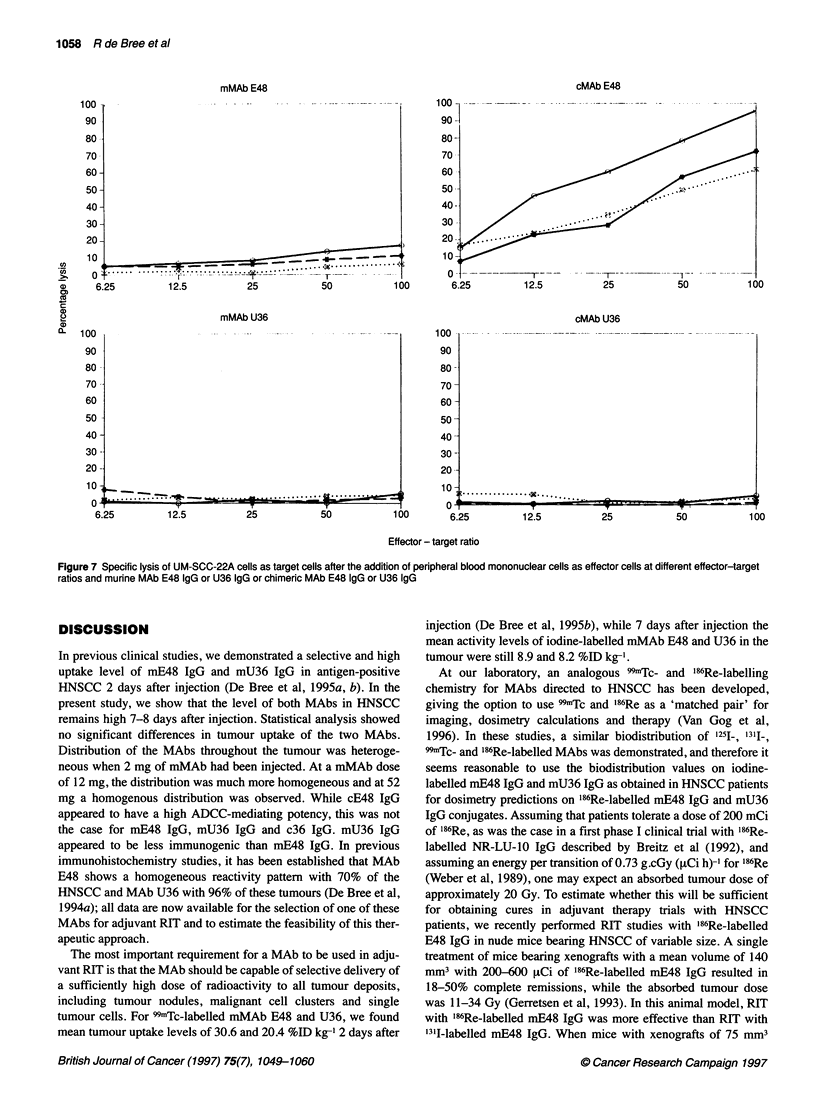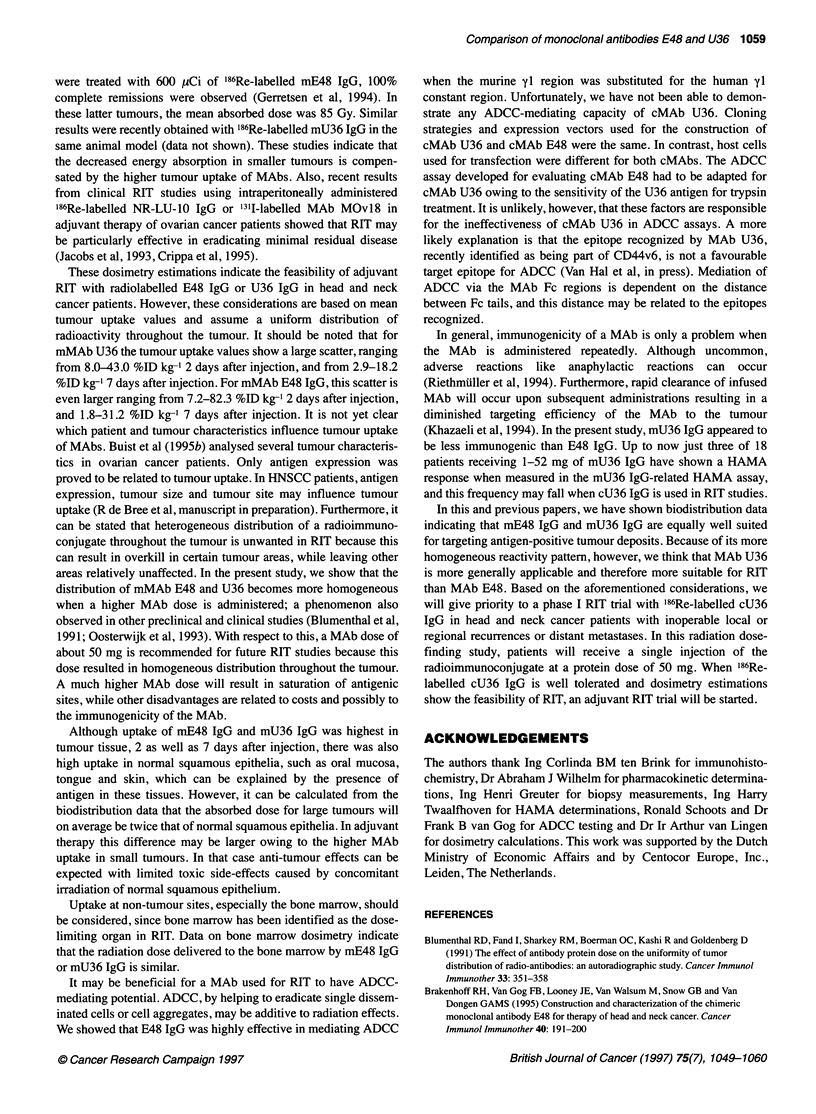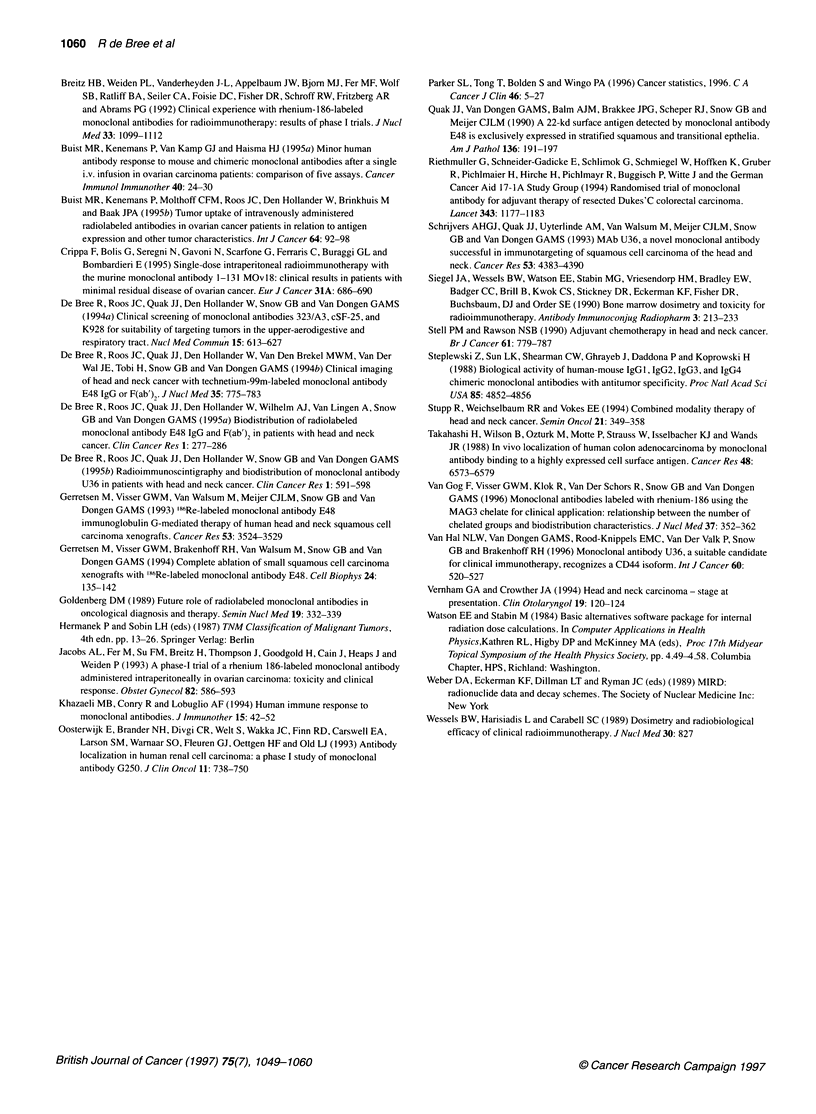# Selection of monoclonal antibody E48 IgG or U36 IgG for adjuvant radioimmunotherapy in head and neck cancer patients.

**DOI:** 10.1038/bjc.1997.179

**Published:** 1997

**Authors:** R. de Bree, J. C. Roos, M. A. Plaizier, J. J. Quak, G. J. van Kamp, W. den Hollander, G. B. Snow, G. A. van Dongen

**Affiliations:** Department of Otolaryngology/Head and Neck Surgery, Free University Hospital, Amsterdam, The Netherlands.

## Abstract

**Images:**


*British Journal of Cancer* (1997) **75**(7), 1049–1060
© 1997 Cancer Research Campaign

### Selection of monoclonal antibody E48 IgG or U36 IgG for adjuvant radioimmunotherapy in head and neck cancer patients

**R de Bree[1], JC Roos[2], MABD Plaizier[2], JJ Quak[1], GJ van Kamp[3], W den Hollander[2], GB Snow[1] and GAMS van Dongen[1]**

Departments of [1]Otolaryngology/Head and Neck Surgery, [2]Nuclear Medicine and [3]Clinical Chemistry, Free University Hospital, Amsterdam, The Netherlands

**Summary** Preliminary data from recent clinical radioimmunoscintigraphy studies indicate that $^{99m}$Tc-labelled murine monoclonal antibodies (MAbs) E48 and U36 have a similar ability to target squamous cell carcinoma of the head and neck (HNSCC) selectively. In the present study we describe additional aspects of murine and chimeric MAb (mMAb and cMAb) E48 and U36, which might influence the selection of one MAb for adjuvant radioimmunotherapy. To make direct comparison possible, ten patients received 11.2 ± 0.3 and 11.1 ± 0.2 mg ($n$ = 5) or 51.1 ± 0.1 and 51.0 ± 0.4 mg ($n$ = 5) of both mE48 IgG and mU36 IgG labelled with $^{131}$I and $^{125}$I simultaneously and underwent surgery 7–8 days after injection. The mean uptake of iodine-labelled mE48 IgG and mU36 was highest in tumour tissue, 8.9 ± 8.9 and 8.2 ± 4.4 %ID kg$^{-1}$ respectively. Tumour to non-tumour ratios for oral mucosa, skin, muscle, blood and bone marrow aspirate were 2.5, 5.5, 25.2, 4.7 and 4.0 respectively in the case of mE48 IgG and 2.3, 4.1, 21.0, 5.8 and 5.8 respectively in the case of mU36 IgG. The distribution of mMAbs E48 and U36 throughout tumours that had been collected in previous studies was heterogeneous when administered at a dose of 1 or 12 mg, and homogeneous when administered at a dose of 52 mg. Administration of mE48 IgG (1–52 mg) resulted in a human anti-mouse antibody response in 12 out of 28 patients, while for mU36 IgG (1–52 mg), this figure was three out of 18 patients. cMAb E48 was shown to be highly effective in mediating antibody-dependent cellular cytotoxicity in vitro, while cMAb U36 and mMAbs E48 and U36 were not effective at all. Rationales are provided that give priority to the start of adjuvant radioimmunotherapy trials with $^{186}$Re-labelled cMAb U36 IgG in head and neck cancer patients who are at high risk for the development of locoregional recurrences and distant metastases.

**Keywords:** monoclonal antibodies; head and neck cancer; squamous cell carcinoma; biodistribution; human anti-mouse antibody response; antibody-dependent cellular cytotoxicity

During 1996 approximately 41 090 Americans will develop head and neck cancer and 12 510 will die from it. Worldwide more than 500 000 new cases are projected annually, and the incidence is rising. In head and neck cancer, squamous cell carcinoma accounts for approximately 90% of all tumours (Parker et al, 1996). About one-third of these patients present with early-stage (I and II) head and neck squamous cell carcinoma (HNSCC), while two-thirds present with advanced disease (stage III and IV) (Vernham and Crowther, 1994). Although early-stage HNSCC can, in the great majority of cases, be cured with surgery or radiotherapy alone, the local failure rate after surgery and/or radiotherapy in advanced stages is more than 50%. Moreover, about 25% of these patients develop distant metastases (Stupp et al, 1994).

Despite an increase in the locoregional control of HNSCC, owing to improved surgery and radiotherapy, current therapy regimens have failed to increase the 5-year survival rate in HNSCC patients (Parker et al, 1996). Whereas fewer patients tend to die from uncontrolled locoregional disease, more patients are exposed to the risk of developing distant metastases. Therefore, an effective systemic adjuvant therapy is needed. To date, there is no evidence that adjuvant chemotherapy has any survival benefit (Stell and Rawson, 1990). Among the innovative approaches for improving the therapy of cancer is the use of monoclonal antibodies (MAbs). MAbs labelled with radionuclides offer the potential of highly localized radiation treatment of cancer (Goldenberg, 1989). Radioimmunotherapy (RIT) may be particularly suitable for the treatment of HNSCC owing to the intrinsic radiosensitivity of this tumour type (Wessels et al, 1989).

For effective adjuvant RIT, it is a requirement that the MAb shows a high and selective uptake as well as retention in all tumour deposits, including tumour nodules, malignant cell clusters and single tumour cells. When β-emitters are used for RIT, it is not necessary that radiolabelled MAbs bind to each single cell within a tumour because of the cross-fire effect. Despite this, heterogeneous distribution of a radioimmunoconjugate throughout the tumour is unwanted in RIT because this can result in overkill in certain tumour areas, while leaving other areas relatively unaffected. When using chimerized, humanized or human MAbs in RIT, it can be anticipated that antibody-dependent cellular cytotoxicity (ADCC) may be supportive to irradiation, especially in eradicating single disseminated cells or small cell aggregates (Riethmüller et al, 1995). For the most efficient mediation of ADCC, these MAbs should preferably contain a human $\gamma 1$ constant region (Steplewski et al, 1988). Such MAbs may also be less immunogenic than murine MAbs, which is of importance when repeated administrations are needed for effective therapy (Khazaeli et al, 1994).

*Received 11 January 1996*
*Revised 1 October 1996*
*Accepted 22 October 1996*

*Correspondence to*: GAMS van Dongen, Department of Otolaryngology/Head and Neck Surgery, Free University Hospital, De Boelelaan 1117, 1081 HV Amsterdam, The Netherlands

For use in adjuvant RIT of HNSCC, we produced a panel of MAbs. Two of these MAbs, designated E48 and U36, are selectively reactive with normal and malignant squamous and transitional epithelia (Quak et al, 1990; Schrijvers et al, 1993). While MAb E48 shows a homogeneous reactivity with 70% of HNSCC, for MAb U36 this approaches 96% reactivity (De Bree et al, 1994*a*). The biodistribution of $^{99m}$Tc-labelled mE48 IgG and mU36 IgG has been evaluated by radioimmunoscintigraphy (RIS) and by biopsy measurement (De Bree et al, 1994*b*, 1995*a*, *b*) in 24 and ten HNSCC patients respectively. Both MAbs were shown to be highly capable of selective tumour targeting in HNSCC patients, and the tumour uptake 2 days after injection was similarly high, 30.6 ± 20.1 %ID kg$^{-1}$ for mMAb E48 and 20.4 ± 12.4 %ID kg$^{-1}$ for mMAb U36 (De Bree et al, 1995*a*,*b*). Most recently, chimeric (mouse/human) E48 IgG$_1$ and U36 IgG$_1$ have been constructed (Brakenhoff et al, 1995).

Data obtained from these studies justify the further development of one of these MAbs for adjuvant RIT. In this paper, we evaluate the potential of these MAbs for adjuvant therapy in a comparative way. To eliminate interindividual variations, mMAbs E48 and U36 were now administered simultaneously. Moreover, we used iodine labels to be able to assess the biodistribution of mMAbs at later time points. We report on (1) the biodistribution of simultaneously injected iodine-labelled mE48 IgG and mU36 IgG, 7–8 days after injection; (2) the distribution of the mMAbs throughout the tumour in relation to the administered MAb dose; (3) the immunogenicity of the mMAbs; and (4) the potential of the murine and chimeric versions of the MAbs to mediate ADCC. Based on our present and previous data, we come to a design for a first RIT trial in head and neck cancer patients.

### MATERIALS AND METHODS

### Monoclonal antibodies

MAb E48 was derived from a mouse immunized with cells from a metastasis of a moderately differentiated squamous cell carcinoma of the larynx. The antigen recognized by MAb E48 is a 16- to 22-kDa glycosylphosphatidylinositol-anchored membrane protein located on the outer cell surface (Quak et al, 1990). The MAb E48-defined antigen was expressed in 94% of the primary HNSCCs ($n$ = 196). In 70% of these tumours, the antigen was expressed by the majority of the cells within these tumours. A similar reactivity pattern was observed in 31 tumour-infiltrated lymph nodes from neck dissection specimens (De Bree et al, 1994*a*). MAb reactivity with normal tissues is restricted to normal stratified squamous epithelium and urothelium of the bladder.

MAb U36 was derived after immunization of mice with viable cells of the HNSCC cell line, UM-SCC-22B. The antigen recognized by MAb U36 is a 200-kDa protein located at the outer cell surface (Schrijvers et al, 1993). The MAb U36-defined antigen is expressed by 99% of the primary HNSCCs ($n$ = 196). In 96% of these tumours, the antigen was expressed by the majority of cells within these tumours. A similar reactivity pattern was observed in 31 lymph node metastases (De Bree et al, 1994*a*). MAb reactivity with normal tissues is restricted to normal stratified squamous epithelium and urothelium of the bladder.

Chimeric (mouse/human) MAbs E48 and U36 containing the human $\gamma 1$ constant region were constructed by recombinant DNA technology as described previously (Brakenhoff et al, 1995).

cSF-25 is the chimeric form of MAb SF-25, which has been developed and characterized by Takahashi et al (1988). The antigen recognized by MAb SF-25 is a 125-kDa protein expressed by adenocarcinomas of the colon and among normal tissues in the distal tubule of the kidney.

### Biodistribution of iodine-labelled mE48 IgG and mU36 IgG simultaneously injected into ten HNSCC patients

The protocol was approved by the Dutch Health Council and by the institutional review board of the Free University Hospital. Informed consent was obtained from all patients. Ten patients with HNSCC were injected with iodine-labelled mE48 IgG and mU36 IgG simultaneously. Before enrolment a biopsy of the primary tumour had to show E48 IgG- and U36 IgG-positive immunoperoxidase staining with more than 75% of the tumour cells. The primary tumour and the status of the neck lymph nodes were classified according to the TNM system of the International Union Against Cancer, the UICC (Hermanek and Sobin, 1987).

All patients received 1.2 ± 0.2 mg of mE48 IgG and 1.1 ± 0.3 mg of mU36 IgG radiolabelled with either $^{131}$I (mean dose 75.8 ± 3.8 MBq) or $^{125}$I (mean dose 3.2 ± 0.5 MBq) by intravenous injection in 5 min. Patient and tumour characteristics and injected MAb dose are listed in Table 1. In five cases, mE48 IgG was labelled with $^{125}$I and mU36 IgG with $^{131}$I. In the other five cases mE48 IgG and mU36 IgG were labelled with $^{131}$I and $^{125}$I respectively. Five

**Table 1** Patient and tumour characteristics and injected MAb doses

| Patient | Age | Sex | Stage[a] | Primary tumour site[a] | E48 IgG dose (mg) | | U36 IgG dose (mg) | |
|---|---|---|---|---|---|---|---|---|
| | | | | | Labelled | Unlabelled | Labelled | Unlabelled |
| 1 | 59 | M | pT3N1 | Oral cavity, lateral tongue | 1.0 | 10.0 | 1.0 | 10.0 |
| 2 | 52 | F | pT3N0 | Oral cavity, floor of mouth | 1.6 | 10.0 | 1.0 | 10.0 |
| 3 | 54 | M | pT3N2b | Oral cavity, lateral tongue | 1.5 | 10.0 | 1.0 | 10.0 |
| 4 | 41 | M | pT3N1 | Oral cavity, lateral tongue | 1.0 | 10.0 | 1.4 | 10.0 |
| 5 | 45 | M | pT3N2b | Oropharynx, tonsil | 1.0 | 10.0 | 1.3 | 10.0 |
| 6 | 50 | M | pT3N2b | Oropharynx, posterior pharyngeal wall | 1.0 | 50.0 | 1.3 | 50.0 |
| 7 | 65 | M | pT2N2b | Oral cavity, lateral tongue | 1.2 | 50.0 | 0.7 | 50.0 |
| 8 | 68 | M | pT3N1 | Oral cavity, lateral tongue | 1.0 | 50.0 | 1.5 | 50.0 |
| 9 | 50 | M | pT4N2b | Oral cavity, retromolar area | 1.3 | 50.0 | 1.0 | 50.0 |
| 10 | 65 | M | pT3N2c | Oral cavity, floor of mouth | 1.0 | 50.0 | 0.7 | 50.0 |

[a] All tumours are squamous cell carcinomas and are staged according to the TNM system of the IUCC.

© Cancer Research Campaign 1997

patients additionally received 10.0 mg, and five patients additionally received 50.0 mg of both unlabelled mE48 IgG and unlabelled mU36 IgG at the time of injection of the radiolabelled MAbs. To prevent uptake of radioactive iodine in the thyroid gland, all patients received Lugol's solution seven droplets three times a day for 7 days starting 1 day preinjection. RIS was performed with a large field of view gamma camera (Dual Head Genesys Imaging system, ADAC Laboratories, Milpitas) equipped with high-energy parallel-hole collimators and connected to a computer (Pegasys, ADAC Laboratories, Malpitas) as described previously (De Bree et al, 1995*b*). Planar anterior and posterior images of the head and neck and whole-body images were obtained up to 8 days post injection

Before and up to 7 days after administration of the radioimmunoconjugates, urine and blood were obtained for analysis as described previously (De Bree et al, 1994*b*). Vital signs were recorded before and up to 3 h after injection.

### *Radiolabelling of mE48 IgG and mU36 IgG*

mE48 IgG and mU36 IgG were prepared, purified and radiolabelled as described previously (De Bree et al, 1995*a*, *b*). The purified mE48 IgG was $^{131}$I- or $^{125}$I-labelled with a specific activity of 79.2 ± 25.7 MBq mg$^{-1}$ or 3.1 ± 0.4 MBq mg$^{-1}$ protein respectively. For mU36 IgG these values were 64.8 ± 19.6 MBq mg$^{-1}$ and 3.5 ± 0.4 MBq mg$^{-1}$ protein respectively. The mean $^{131}$I and $^{125}$I incorporation percentages were 98.5 ± 1.3% and 97.1 ± 2.2%, respectively, as determined by chromatography on ITLC-SG strips (Gelman Sciences, Ann Arbor, MI, USA) with 0.1 M citrate buffer, pH 5.0. As determined by a modified Lineweaver–Burke plot, the immunoreactive fractions of $^{131}$I- or $^{125}$I-labelled mE48 IgG and mU36 IgG at infinite antigen excess always exceeded 68%. The affinity constants were $1.2 \times 10^{10}$ M$^{-1}$ for mE48 IgG and $3.5 \times 10^{10}$ M$^{-1}$ for mU36 IgG as determined by Scatchard analysis.

### *Biodistribution of MAbs 7–8 days after injection*

Biopsies of the primary tumour and several other tissues were taken from the surgical specimens of all patients. Blood, bone marrow aspiration and bone marrow biopsy (containing bone and bone marrow) were taken under general anaesthesia just before surgery. All biopsies were weighed and the amounts of $^{125}$I and $^{131}$I were measured by differential counting methods in a well-counter (1282 Compugamma, LKB Wallac, Turku, Finland) to compare biodistribution of mE48 IgG and mU36 IgG. The effect of self-absorption by volume effects was corrected by comparison of the sample with a set of reference samples, prepared by diluting an equal amount of the standard in different volumes of saline. All data were corrected for decay and converted to percentage injected dose per kilogram (%ID kg$^{-1}$) tissue. If in a patient several biopsies of one kind of tissue were taken, the mean uptake in this tissue was calculated and used for further analysis. Tumour to non-tumour ratios were calculated using matched uptake values of each patient. After counting, all biopsies were analysed histopathologically to determine the presence or absence of HNSCC.

### *Pharmacokinetics*

Blood samples were obtained from the arm opposite to the injection site for the determination of activity up to 7–8 days p.i. Urine was collected over the first 48 h. Aliquots of blood and urine samples were measured for $^{131}$I and $^{125}$I activity in a well counter, compared with an aliquot retained from the conjugate preparation and corrected for decay. Blood activity was expressed as %ID kg$^{-1}$. High-performance liquid chromatography (HPLC) analysis of the serum samples revealed that more than 95% of the radioactivity was bound to the MAb. The pharmacokinetics was analysed modelling a time vs radioactivity curve for each infusion as described previously (De Bree et al, 1995*a*,*b*). Excretion in the urine was expressed as the percentage of the injected dose in this period.

### *Bone marrow dosimetry*

Blood and whole-body data of both the $^{125}$I- and $^{131}$I-labelled mE48 IgG and mU36 IgG were used to calculate the marrow dose. The residence times of mMAb E48 and mMAb U36 in the blood and whole body were derived from numerical integration of the blood and urinary time activity curves. The blood and whole-body time activity curves were extrapolated to infinity using the decay factor between the last two measurement points. The activity in the whole body was assumed to be homogeneously distributed. No specific uptake in bone marrow was observed by RIS during the first days after injection. Therefore, the red marrow–blood activity ratio was assumed to be 0.3 (Siegel et al, 1990). The red marrow dose was calculated according to MIRDDOSE2 (Watson and Stabin, 1984). The red marrow dose was estimated from the kinetic data and a $^{131}$I label.

### *Statistical analysis*

The Student's *t*-test for paired and unpaired data was used to determine the statistical significance of the difference between the uptake of mE48 IgG and mU36 IgG. Statistical difference was reached at $P < 0.05$.

### Further characteristics of E48 IgG and U36 IgG

To obtain more information on the characteristics of E48 IgG and U36 IgG, data from this and previous studies with radiolabelled E48 IgG or U36 IgG alone were combined. The patient populations from previous studies have been described before (De Bree et al, 1994*b*, 1995*a*,*b*). In these studies, 19 patients received mE48 F(ab')$_2$, nine patients received mE48 IgG, 15 patients received mE48 F(ab')$_2$ as well as mE48 IgG, and ten patients received mU36 IgG.

### *Distribution of the MAbs throughout the tumour*

Cryosections were made from tumour biopsies obtained from patients included in previous studies (De Bree et al, 1995*a*,*b*). These biopsies had been taken 2 days after injection of either mE48 IgG or mU36 IgG. The in vivo distribution of the injected MAb throughout the tumour was compared with the maximal MAb binding on serial sections. In short, 5-$\mu$m-thick sections of frozen tissue biopsies were cut on a cryostat microtome and mounted on poly-L-lysine-coated glass slides, dried, fixed in 2% paraformaldehyde in phosphate-buffered saline (PBS) for 10 min and dried again.

To assess the distribution of the injected MAb throughout the tumour, the specimens were incubated for 30 min with rabbit anti-mouse IgG (Dako, Glostrup, Denmark) diluted 1:25 in PBS/1% normal rabbit serum/1% bovine serum albumin (BSA). The sections were washed three times with PBS and incubated for 1 h with alkaline phosphatase monoclonal anti-alkaline phosphatase (APAAP, Dako) diluted in PBS/1% BSA. The sections were washed again with PBS/1% BSA, rinsed with 0.1 M Tris, pH 8.2, and incubated in the dark for 20 min with naphthol AS-TR-phosphate/fast red violet

© Cancer Research Campaign 1997

LB substrate (both from Sigma). The substrate was prepared as follows: 6 mg naphthol AS-TR-phosphate dissolved in 250 μl of dimethylformamide was added to 10 ml of 0.1 M Tris-HCl, pH 8.2, containing 10 μl of 1 M levamisole. Immediately before this preparation was used, 10 mg of fast red violet LB was added and the solution was filtered. Sections were washed for 1 min with running tap-water and counterstained with haematoxylin for 45 s, dehydrated and covered with Kaiser's glycerol gelatin (Merck, Amsterdam, The Netherlands).

Maximum MAb binding was assessed in the same way, except that the specimens were incubated with undiluted hybridoma culture supernatant containing the MAb corresponding to the MAb used for injection. Isotype-matched control MAbs and PBS served as negative controls.

### *Human anti-mouse antibody (HAMA) responses*

***Patients*** In the present study, all data on HAMA responses of patients included in the present and previous RIS studies (De Bree et al, 1994b, 1995a,b), and injected with mE48 $F(ab')_2$, mE48 IgG, mU36 IgG or combinations thereof, are presented. The presence of human anti-mouse antibodies was tested in patients' sera before and 4–8 weeks after injection of radiolabelled mE48 $F(ab')_2$ (group 1, n = 16); mE48 IgG (group 2, n = 5); mE48 $F(ab')_2$ plus mE48 IgG (group 3, n = 13); mU36 IgG (group 4, n = 8); or mE48 IgG plus mU36 IgG (group 5, n = 10).

Fourteen patients in group 1 received 1 mg and two patients received 11 mg of mE48 $F(ab')_2$. The five patients of group 2 received 1 mg of mE48 IgG. Ten patients in group 3 received 2 mg, one patient 12 mg and two patients 52 mg of mE48 IgG simultaneously with 1 mg of mE48 $F(ab')_2$. Four patients in group 4 received 2 mg, two patients 12 mg and two patients 52 mg of mU36 IgG. Five patients in group 5 simultaneously received 11 mg of mE48 IgG and 11 mg of mU36 IgG, while five patients simultaneously received 51 mg of mE48 IgG and 51 mg of mU36 IgG.

***Assay*** HAMA responses were analysed with an assay, essentially as described previously (Buist et al, 1995*a*); briefly, murine MAbs E48 $(Fab')_2$, E48 IgG or U36 IgG (20 pg per well) were bound to the wells of a microtitre plate, coated with goat polyclonal anti-mouse IgG antibodies (Dako Z0420). Patients' sera were prediluted stepwise: 1:50, 1:200, 1:800 and 1:3200, and incubated at 100 μl per well in duplicate. As controls, a negative serum was diluted 1:50 and a positive serum 1:1500. Any human IgG bound was detected with horse-radish peroxidase-labelled rabbit anti-human IgG (Dako P0212) and *o*-phenylenediamine as substrate. Reactions were performed at room temperature, until the absorbance of the control serum wells reached a value of approximately 1.0. The reaction was stopped by adding 50 μl of 4N sulphuric acid, and the extinction was measured at 490 nm in a plate reader. The reciprocal serum dilution yielding an absorbance reading of 1.0 is called the HAMA titre and is calculated from the formula: HAMA titre = $(A-1)\times(Z-Y)/(A-B) + Y$, where $Y$ is the serum dilution corresponding to the nearest optical density ($A$) above 1.0 and $Z$ is the serum dilution corresponding to the nearest optical density ($B$) below 1.0. A HAMA titre of ≥ 500 was arbitrarily considered to be positive. HAMA titres provided are the mean of duplicate or triplicate analyses. Standard errors of the mean have been omitted, as they were less than 8%. Sera obtained from patients injected with a combination of mE48 $F(ab')_2$ and IgG were analysed with a mE48 $F(ab')_2$- and a mE48 IgG-related HAMA assay, which means that mE48 $F(ab')_2$ and mE48 IgG was used as catcher respectively. Analagously, mE48 IgG- and mU36 IgG-related HAMA assays were used for the analysis of sera obtained from patients who had received mE48 IgG and mU36 IgG simultaneously.

### *Antibody-dependent cellular cytotoxicity*

UM-SCC-22A cells served as target cells, and peripheral blood mononuclear cells (PBMCs) were obtained from healthy volunteers as effector cells in ADCC assays. PBMCs were obtained as described previously (Brakenhoff et al, 1995)

Target cells were trypsinized with 0.05% trypsin, 0.02% EDTA in PBS (Gibco Life Technologies), washed with tissue culture medium and resuspended in tissue culture medium at a concentration of $10^5$ cells $ml^{-1}$. For ADCC assays with mMAb E48 or cMAb E48, 2.5 μCi of sodium $[^{51}Cr]$chromate was immediately added together with $5 \times 10^3$ cells (50 μl) in the wells of 96-well U-bottomed plates (Greiner, Westmalle, Belgium). Since the reactivity of MAb U36 with UM-SCC-22A cells is lost upon trypsinization of these cells, the ADCC assay was slightly modified for testing the ADCC-mediating activity of mMAb U36 and cMAb U36. In this case, UM-SCC-22A cells were incubated for 48 h in 96-well U-bottomed plates before 2.5 μCi of $^{51}Cr$ was added, thus allowing total recovery of U36 antigen expression and MAb U36 binding [as assessed by enzyme-linked immunosorbent assay (ELISA) methods]. At the moment of $^{51}Cr$ addition, each well contained $5 \times 10^3$ target cells.

After incubation of the cells for 16 h with $^{51}Cr$ in 5% carbon dioxide at 37°C, the cells were washed three times with Dulbecco's modified Eagle medium (DMEM)/5% FCS, effector cells were added at different effector–target (E:T) ratios ranging from 6.25:1 to 100:1 and MAbs were added to a final concentration of 10 μg $ml^{-1}$. Plates were centrifuged at 65 × ***g*** for 30 s and incubated in 5% carbon dioxide at 37°C for 5 h after which medium was harvested from each well. All assays were performed in triplicate. Radioactivity was counted in a gamma-counter (1282 CompuGamma). Maximum radionuclide release was determined by incubation of target cells in the presence of 5% Triton X-100. Natural killer (NK) cell release (antibody-independent lysis) was measured by incubation of target–effector cells in the presence of the murine $IgG_1$ control anti-myosin MAb Myoscint (Centocor Inc.), which does not bind to the target cells. The percentage of specific lysis was calculated as the [(experimental release–background release)/(Triton release–background release)] × 100. Background release is defined as the release in the absence of specific antibody (= spontaneous release + NK release). Standard deviations were typically 0–8%.

### RESULTS

### Biodistribution of iodine-labelled mE48 and mU36 IgG simultaneously injected into ten HNSCC patients

No adverse reactions were observed that could be related to the simultaneous injection of iodine-labelled mMAb E48 and mMAb U36. No significant changes were noted in blood and urine parameters except for TSH values. In three patients, TSH was found transiently below normal after injection. In one of these patients, TSH was already at the lower limit of normal before injection. Free T3 increased in two of these patients to high levels. However,

© Cancer Research Campaign 1997

**Figure 1** Anterior (**A**) and posterior (**B**) whole-body images of patient 3, 3 days after injection of $^{131}$I-labelled mE48 IgG. Note the visualization of the lateral tongue carcinoma on the left side (arrow)

**Figure 2** Anterior (**A**) and posterior (**B**) whole-body images of patient 8, 4 days after injection of $^{131}$I-labelled mU36 IgG. Note the visualization of the lateral tongue carcinoma on the left side (arrow)

the patients remained clinically euthyroid and FT4 and total T3 levels remained within normal limits. In those two patients the thyroid was visualized. Further analyses will be performed to find out whether these changes in TSH are related, for example, to the administration of Lugol's solution, and the changes in T3 to the administration of MAbs and experimental artifacts in the assay systems used.

**Table 2** Uptake in %ID kg$^{-1}$ of simultaneously injected iodine-labelled E48 IgG and U36 IgG for individual patients in tumour biopsies obtained from the surgical specimens 7–8 days after injection

| Patient | $^{125}$I/$^{131}$I-labelled E48 | $^{131}$I/$^{125}$I-labelled U36 |
|---|---|---|
| 1 | 1.8 | 2.9 |
| 2 | 3.1 | 5.2 |
| 3 | 8.2 | 9.4 |
| 4 | 31.2 | 13.7 |
| 5 | 3.3 | 3.7 |
| 6 | 2.7 | 4.1 |
| 7 | 2.1 | 4.6 |
| 8 | 10.7 | 12.6 |
| 9 | 8.2 | 10.9 |
| 10 | 18.2 | 15.2 |

### *Radioimmunoscintigraphy with mMAbs E48 and U36*

Whole-body images were obtained up to 3 ($n$ = 2), 4 ($n$ = 4), 6 ($n$ = 2) and 8 ($n$ = 1) days p.i. One patient did not comply with the imaging protocol and was lost to follow-up. No selective targeting outside the head and neck region was observed (Figures 1 and 2). The tracer was homogeneously distributed in the area of the liver, spleen and kidneys and, in some patients with both mMAb E48 and mMAb U36, the lungs showed faint uptake. The scrotal area was visualized with mMAb E48 as well as with mMAb U36. The skeleton/bone marrow was not visualized. In six out of nine patients, the primary tumour was visualized. The quality of the images was insufficient to detect lymph node metastases owing to the use of a relative low dose of $^{131}$I.

### *Biodistribution of mMAbs E48 and U36, 7–8 days after injection*

The activity uptake in biopsies from the surgical specimen is shown in Tables 2 and 3. The mean uptake of iodine was the highest in tumour tissue: 8.9 ± 8.9 %ID kg$^{-1}$ for iodine-labelled mE48 IgG and 8.2 ± 4.4 %ID kg$^{-1}$ for iodine-labelled mU36 IgG. Table 2 shows that the variability in tumour uptake is smaller for mMAb U36 (range 2.9–15.2 %ID kg$^{-1}$) than for mMAb E48 (range 1.8–31.2 %ID kg$^{-1}$). Moreover, it appears that in eight out of ten patients the tumour uptake of mU36 IgG was higher than the uptake of mE48 IgG.

High uptake of the mMAb E48 and the mMAb U36 iodine labels was also seen in normal squamous epithelium of oral mucosa, tongue tissue and skin. These uptake levels were lower than in tumour tissue, but only significantly for mU36 IgG uptake in mucosa and tongue ($P$ < 0.01 and $P$ < 0.02 respectively). Tumour-positive lymph nodes contained more iodine than tumour-negative nodes for mE48 IgG ($P$ < 0.2, mean ratio 2.0 ± 1.1) and mU36 IgG ($P$ < 0.01, mean ratio 3.0 ± 1.2). Iodine uptake in other normal tissues was low, except for iodine-labelled mU36 IgG in glandular tissue. Low levels of activity were seen in bone marrow biopsies. Bone marrow aspirations showed mean iodine levels which were similar for mE48 IgG and mU36 IgG and almost similar to the activity levels in blood (mean bone marrow aspirate – blood ratios of 0.9 ± 0.0 and 1.0 ± 0.1 in the case of mE48 IgG and mU36 IgG respectively). The activity in the plasma of the bone marrow aspirate was higher than in the cellular sediment. The mean plasma activity in the bone marrow aspirate was slightly lower than in blood plasma; mean bone marrow aspirate plasma–blood plasma ratios were 0.9 ± 0.3 and 1.0 ± 0.1 for mE48 IgG and mU36 IgG respectively.

© Cancer Research Campaign 1997

**Table 3** Uptake of simultaneously injected iodine-labelled E48 IgG and U36 IgG in biopsies obtained from the surgical specimens 7–8 days after injection

| Tissue | $^{125/131}$I-labelled E48 IgG (%ID kg$^{-1}$) Mean ± s.d. | Range | $^{131/125}$I-labelled U36 IgG (%ID kg$^{-1}$) Mean ± s.d. | Range | $n$[a] |
|---|---|---|---|---|---|
| Tumour | 8.9 ± 8.9 | 1.8–31.2 | 8.2 ± 4.4 | 2.9–15.2 | 10 |
| Mucosa | 4.1 ± 1.4 | 2.0–6.8 | 4.0 ± 1.4 | 1.6–5.8 | 10 |
| Tongue | 5.8 ± 2.7 | 1.8–8.8 | 4.4 ± 2.3 | 1.3–7.9 | 5 |
| Skin | 2.5 ± 1.0 | 1.3–4.2 | 2.5 ± 1.0 | 1.5–4.2 | 5 |
| Positive lymph node | 1.3 ± 1.2 | 0.4–4.6 | 2.0 ± 1.1 | 0.5–4.6 | 9 |
| Negative lymph node | 0.6 ± 0.3 | 0.3–1.3 | 0.7 ± 0.4 | 0.3–1.4 | 10 |
| Muscle | 0.4 ± 0.2 | 0.2–0.7 | 0.4 ± 0.2 | 0.2–1.0 | 10 |
| Fat | 0.3 ± 0.2 | 0.2–0.7 | 0.4 ± 0.2 | 0.1–1.0 | 10 |
| Submandibular gland | 1.1 ± 0.6 | 0.6–2.5 | 3.9 ± 1.2 | 2.0–5.8 | 10 |
| Sublingual gland | 0.2 | | 1.4 | | 1 |
| Vein | 0.7 ± 0.3 | 0.4–1.1 | 0.8 ± 0.3 | 0.3–1.4 | 7 |
| Bone marrow biopsy | 0.4 ± 0.2 | 0.2–0.7 | 0.5 ± 0.2 | 0.2–0.7 | 6 |
| Total bone marrow aspiration | 1.2 ± 0.4 | 0.7–1.7 | 1.5 ± 0.5 | 0.4–1.8 | 6 |
| Supernatant bone marrow aspiration | 1.7 ± 0.7 | 0.7–2.9 | 2.1 ± 0.9 | 0.5–3.0 | 6 |
| Sediment bone marrow aspiration | 0.8 ± 0.9 | 0.3–2.9 | 0.5 ± 0.2 | 0.2–0.7 | 6 |
| Blood | 1.4 ± 0.4 | 0.8–1.9 | 1.5 ± 0.6 | 0.3–2.7 | 9 |
| Plasma | 2.1 ± 0.5 | 1.3–2.9 | 2.3 ± 0.8 | 0.6–3.8 | 9 |

[a] Number of patients from whom biopsies were obtained.

**Figure 3** Primary tumour to non-tumour ratios of simultaneously injected iodine-labelled mE48 IgG (■) and mU36 (□) IgG in mucosa (Mc; $n$ = 10), tongue (To; $n$ = 5), skin (Sk; $n$ = 5), positive lymph node (PL; $n$ = 9), negative lymph node (NL; $n$ = 10), muscle (Ms; $n$ = 10), fat (Ft; $n$ = 10), submandibular gland (Sm; $n$ = 10), sublingual gland (Sl; $n$ = 1), vein (Ve; $n$ = 7), bone marrow biopsy (BB; $n$ = 6), total bone marrow aspiration (BA; $n$ = 6), supernatant of bone marrow aspiration (Su; $n$ = 6), sediment of bone marrow aspiration (Se; $n$ = 6), blood (Bl; $n$ = 9) and plasma (Pl; $n$ = 9)

For iodine-labelled mE48 IgG, the mean tumour to non-tumour ratios varied between 1.8 for tongue and 34.2 for fat tissue, while for iodine-labelled mU36 IgG these values varied between 2.1 for sublingual gland and 28.7 for fat tissue (Figure 3). For mucosa, tongue, skin, submandibular gland, muscle, blood and bone marrow aspirate these ratios were 2.5, 1.8, 5.5, 9.8, 25.2, 4.7 and 4.0, respectively for mE48 IgG and 2.3, 2.3, 4.1, 2.4, 21.0, 5.8 and 5.8, respectively for mU36 IgG. Increase of MAb doses from 11–51 mg did not alter the activity levels in tissues or the tumour to non-tumour ratios. There were no significant differences in uptake values or tumour to non-tumour ratios between mE48 IgG and mU36 IgG, except for the uptake in glandular tissue ($P < 0.001$).

### *Pharmacokinetics*

The time vs radioactivity curves of iodine-labelled mE48 IgG and mU36 IgG best fit a two-compartment model. There was no significant difference in elimination from the blood for iodine-labelled

*© Cancer Research Campaign 1997*

**Figure 4** Distribution of mE48 IgG throughout tumour biopsies obtained from HNSCC patients 2 days after injection of 2 mg (**A** and **B**), 12 mg (**C** and **D**) and 52 mg (**E** and **F**). **A**, **C** and **E** represent MAb distribution as assessed by immunohistochemical staining with rabbit anti-mouse IgG. **B, D** and **E** represent antigen expression (maximum MAb binding) as assessed by immunohistochemistry with mE48 IgG followed by rabbit anti-mouse IgG. Note the more homogeneous distribution at higher dose, reaching binding to all tumour cells at 52 mg of mMAb E48

© Cancer Research Campaign 1997

**Figure 5** Distribution of mU36 IgG throughout a tumour-infiltrated lymph node 2 days after injection of 52 mg of mU36 IgG. **A** represents MAb distribution as assessed by immunohistochemical staining with rabbit anti-mouse IgG. **B** represents antigen expression (maximum MAb binding) as assessed by immunohistochemistry with mU36 IgG followed by rabbit anti-mouse IgG. Note the binding of mU36 IgG to all tumour cells in this lymph node metastasis mE48 IgG and mU36 IgG: $t_{1/2\alpha}$ and $t_{1/2\beta}$ were 5.1 ± 5.3 and 81.3 ± 81.6 h for mE48 IgG and 6.1 ± 3.0 and 66.1 ± 29.5 h for mU36 IgG respectively.

There was also no significant difference in the excretion of iodine-labelled mE48 IgG and mU36 IgG in urine during the first 48 h: 23.0 ± 6.0 and 21.4 ± 5.1% of the injected dose respectively.

### *Bone marrow dosimetry*

Red marrow residence time and whole-body residence time were 4.1 ± 1.5 h and 85.5 ± 21.5 h respectively for mE48 IgG, and 4.3 ± 1.9 h and 88.5 ± 20.6 h respectively for mU36 IgG. The red marrow dose was calculated to be 1.89 ± 0.59 rad $mCi^{-1}$ for $^{131}I$-labelled mE48 IgG and 2.00 ± 0.75 for $^{131}I$-labelled mU36 IgG.

### Further characteristics of E48 IgG AND U36 IgG

### *Distribution of the mMAbs E48 and U36 throughout the tumour*

Distribution of MAbs throughout the tumour was analysed immunohistochemically in tumour biopsies obtained from patients who had been injected with either mE48 IgG or mU36 IgG alone (previous studies). Figure 4 shows typical mE48 IgG uptake in tumour biopsy specimens of three patients who had received 2, 12 and 52 mg of E48 IgG. Serial sections of each biopsy were incubated with rabbit anti-mouse IgG to demonstrate in vivo tumour uptake of mE48 IgG (sections A, C and E), or with mMAb E48 followed by rabbit anti-mouse IgG to demonstrate total E48 antigen expression, the maximum mE48 IgG binding (sections B, D and F). After injection of 2 mg, a very heterogeneous distribution of mE48 IgG was observed. After injection of 12 mg of MAb, the distribution was much more homogeneous, while after injection of 52 mg almost all tumour cells were targeted by mE48 IgG. A similar distribution pattern was observed for mU36 IgG as illustrated for a tumour-infiltrated lymph node obtained from a patient who had received 52 mg of U36 IgG (Figure 5). mE48 IgG or mU36 IgG could be detected in all tumour biopsies of patients who had received 12 or 52 mg of MAb; also in biopsies from tumour deposits not detected by RIS.

### *Immunogenicity of mMAbs E48 and U36*

To assess the immunogenicity of MAbs mE48 and mU36, serum samples obtained from patients before and 4–8 weeks after injection of mE48 $F(ab')_2$ (group 1), mE48 IgG (group 2), mE48 $F(ab')_2$ plus mE48 IgG (group 3), mU36 IgG (group 4) and mE48 IgG plus mU36 IgG (group 5) were analysed for the presence of human anti-mouse antibodies (HAMA) with an in-house mE48 $F(ab')_2$-, mE48 IgG- and/or mU36 IgG-related HAMA assay. Considering a HAMA titre of ≥ 500 to be positive, preMAb infusion samples were negative (HAMA titres < 50) for all patients (Figure 6).

© Cancer Research Campaign 1997

**Figure 6** Individual human anti-mouse antibody (HAMA) titres before and 4–8 weeks after injection of (**A**) mE48 F(ab′)$_2$, (**B**) mE48 IgG, (**C**) mE48 F(ab′)$_2$ plus mE48 IgG, (**D**) mU36 IgG and (**E**) mE48 IgG plus mU36 IgG at different MAb doses

Three out of 16 patients (19%) who had received 1 mg ($n$ = 14) or 11 mg ($n$ = 2) of mE48 F(ab′)$_2$ alone showed a HAMA response as measured in the F(ab′)$_2$ related HAMA assay. The highest titre found was 811. Two out of five patients (40%) who had received 1 mg of mE48 IgG alone showed an anti-E48 IgG response with titres of 1558 and 4154 respectively. Ten out of 13 patients (77%) who received a cocktail of 1 mg of mE48 F(ab′)$_2$ and 1–51 mg of mE48 IgG showed a HAMA response with titres up to 3709, when measured in the E48 IgG-related HAMA assay. When the same sera were analysed with the F(ab′)$_2$-related HAMA assay, seven out of 13 appeared to be positive.

HAMA responses were also observed in three out of eight patients (37%) who had received 2–52 mg of mU36 IgG. None of the ten patients who received 11 or 51 mg of mE48 IgG and mU36 IgG simultaneously showed a HAMA response when measured in the mE48 IgG- or mU36 IgG-related HAMA assay.

### *ADCC-mediating potential of mMAbs and cMAbs E48 and U36*

ADCC assays with MAbs E48 and U36 revealed that spontaneous chromium release never exceeded 20%, while antibody-independent killing was less than 10%. When cMAb E48 was included in this assay at an effector–target ratio of 100:1, specific lysis percentages of 62%, 72% and 96% were found respectively (Figure 7). For cMAb U36 these values were 1.2%, 5.2% and 3.5%, for mMAb E48 6%, 11% and 17% and for mMAb U36 5.4%, 3.0% and 4.7%. cMAb SF-25 served as a positive control in each ADCC assay and gave specific lysis percentages between 63% and 78% (data not shown).

© Cancer Research Campaign 1997 

**Figure 7** Specific lysis of UM-SCC-22A cells as target cells after the addition of peripheral blood mononuclear cells as effector cells at different effector–target ratios and murine MAb E48 IgG or U36 IgG or chimeric MAb E48 IgG or U36 IgG

### DISCUSSION

In previous clinical studies, we demonstrated a selective and high uptake level of mE48 IgG and mU36 IgG in antigen-positive HNSCC 2 days after injection (De Bree et al, 1995*a*, *b*). In the present study, we show that the level of both MAbs in HNSCC remains high 7–8 days after injection. Statistical analysis showed no significant differences in tumour uptake of the two MAbs. Distribution of the MAbs throughout the tumour was heterogeneous when 2 mg of mMAb had been injected. At a mMAb dose of 12 mg, the distribution was much more homogeneous and at 52 mg a homogenous distribution was observed. While cE48 IgG appeared to have a high ADCC-mediating potency, this was not the case for mE48 IgG, mU36 IgG and c36 IgG. mU36 IgG appeared to be less immunogenic than mE48 IgG. In previous immunohistochemistry studies, it has been established that MAb E48 shows a homogeneous reactivity pattern with 70% of the HNSCC and MAb U36 with 96% of these tumours (De Bree et al, 1994*a*); all data are now available for the selection of one of these MAbs for adjuvant RIT and to estimate the feasibility of this therapeutic approach.

The most important requirement for a MAb to be used in adjuvant RIT is that the MAb should be capable of selective delivery of a sufficiently high dose of radioactivity to all tumour deposits, including tumour nodules, malignant cell clusters and single tumour cells. For $^{99m}$Tc-labelled mMAb E48 and U36, we found mean tumour uptake levels of 30.6 and 20.4 %ID kg$^{-1}$ 2 days after injection (De Bree et al, 1995*b*), while 7 days after injection the mean activity levels of iodine-labelled mMAb E48 and U36 in the tumour were still 8.9 and 8.2 %ID kg$^{-1}$.

At our laboratory, an analogous $^{99m}$Tc- and $^{186}$Re-labelling chemistry for MAbs directed to HNSCC has been developed, giving the option to use $^{99m}$Tc and $^{186}$Re as a 'matched pair' for imaging, dosimetry calculations and therapy (Van Gog et al, 1996). In these studies, a similar biodistribution of $^{125}$I-, $^{131}$I-, $^{99m}$Tc- and $^{186}$Re-labelled MAbs was demonstrated, and therefore it seems reasonable to use the biodistribution values on iodine-labelled mE48 IgG and mU36 IgG as obtained in HNSCC patients for dosimetry predictions on $^{186}$Re-labelled mE48 IgG and mU36 IgG conjugates. Assuming that patients tolerate a dose of 200 mCi of $^{186}$Re, as was the case in a first phase I clinical trial with $^{186}$Re-labelled NR-LU-10 IgG described by Breitz et al (1992), and assuming an energy per transition of 0.73 g.cGy (μCi h)$^{-1}$ for $^{186}$Re (Weber et al, 1989), one may expect an absorbed tumour dose of approximately 20 Gy. To estimate whether this will be sufficient for obtaining cures in adjuvant therapy trials with HNSCC patients, we recently performed RIT studies with $^{186}$Re-labelled E48 IgG in nude mice bearing HNSCC of variable size. A single treatment of mice bearing xenografts with a mean volume of 140 mm$^3$ with 200–600 μCi of $^{186}$Re-labelled mE48 IgG resulted in 18–50% complete remissions, while the absorbed tumour dose was 11–34 Gy (Gerretsen et al, 1993). In this animal model, RIT with $^{186}$Re-labelled mE48 IgG was more effective than RIT with $^{131}$I-labelled mE48 IgG. When mice with xenografts of 75 mm$^3$

© Cancer Research Campaign 1997

were treated with 600 μCi of $^{186}$Re-labelled mE48 IgG, 100% complete remissions were observed (Gerretsen et al, 1994). In these latter tumours, the mean absorbed dose was 85 Gy. Similar results were recently obtained with $^{186}$Re-labelled mU36 IgG in the same animal model (data not shown). These studies indicate that the decreased energy absorption in smaller tumours is compensated by the higher tumour uptake of MAbs. Also, recent results from clinical RIT studies using intraperitoneally administered $^{186}$Re-labelled NR-LU-10 IgG or $^{131}$I-labelled MAb MOv18 in adjuvant therapy of ovarian cancer patients showed that RIT may be particularly effective in eradicating minimal residual disease (Jacobs et al, 1993, Crippa et al, 1995).

These dosimetry estimations indicate the feasibility of adjuvant RIT with radiolabelled E48 IgG or U36 IgG in head and neck cancer patients. However, these considerations are based on mean tumour uptake values and assume a uniform distribution of radioactivity throughout the tumour. It should be noted that for mMAb U36 the tumour uptake values show a large scatter, ranging from 8.0–43.0 %ID $kg^{-1}$ 2 days after injection, and from 2.9–18.2 %ID $kg^{-1}$ 7 days after injection. For mMAb E48 IgG, this scatter is even larger ranging from 7.2–82.3 %ID $kg^{-1}$ 2 days after injection, and 1.8–31.2 %ID $kg^{-1}$ 7 days after injection. It is not yet clear which patient and tumour characteristics influence tumour uptake of MAbs. Buist et al (1995*b*) analysed several tumour characteristics in ovarian cancer patients. Only antigen expression was proved to be related to tumour uptake. In HNSCC patients, antigen expression, tumour size and tumour site may influence tumour uptake (R de Bree et al, manuscript in preparation). Furthermore, it can be stated that heterogeneous distribution of a radioimmunoconjugate throughout the tumour is unwanted in RIT because this can result in overkill in certain tumour areas, while leaving other areas relatively unaffected. In the present study, we show that the distribution of mMAb E48 and U36 becomes more homogeneous when a higher MAb dose is administered; a phenomenon also observed in other preclinical and clinical studies (Blumenthal et al, 1991; Oosterwijk et al, 1993). With respect to this, a MAb dose of about 50 mg is recommended for future RIT studies because this dose resulted in homogeneous distribution throughout the tumour. A much higher MAb dose will result in saturation of antigenic sites, while other disadvantages are related to costs and possibly to the immunogenicity of the MAb.

Although uptake of mE48 IgG and mU36 IgG was highest in tumour tissue, 2 as well as 7 days after injection, there was also high uptake in normal squamous epithelia, such as oral mucosa, tongue and skin, which can be explained by the presence of antigen in these tissues. However, it can be calculated from the biodistribution data that the absorbed dose for large tumours will on average be twice that of normal squamous epithelia. In adjuvant therapy this difference may be larger owing to the higher MAb uptake in small tumours. In that case anti-tumour effects can be expected with limited toxic side-effects caused by concomitant irradiation of normal squamous epithelium.

Uptake at non-tumour sites, especially the bone marrow, should be considered, since bone marrow has been identified as the dose-limiting organ in RIT. Data on bone marrow dosimetry indicate that the radiation dose delivered to the bone marrow by mE48 IgG or mU36 IgG is similar.

It may be beneficial for a MAb used for RIT to have ADCC-mediating potential. ADCC, by helping to eradicate single disseminated cells or cell aggregates, may be additive to radiation effects. We showed that E48 IgG was highly effective in mediating ADCC when the murine γ1 region was substituted for the human γ1 constant region. Unfortunately, we have not been able to demonstrate any ADCC-mediating capacity of cMAb U36. Cloning strategies and expression vectors used for the construction of cMAb U36 and cMAb E48 were the same. In contrast, host cells used for transfection were different for both cMAbs. The ADCC assay developed for evaluating cMAb E48 had to be adapted for cMAb U36 owing to the sensitivity of the U36 antigen for trypsin treatment. It is unlikely, however, that these factors are responsible for the ineffectiveness of cMAb U36 in ADCC assays. A more likely explanation is that the epitope recognized by MAb U36, recently identified as being part of CD44v6, is not a favourable target epitope for ADCC (Van Hal et al, in press). Mediation of ADCC via the MAb Fc regions is dependent on the distance between Fc tails, and this distance may be related to the epitopes recognized.

In general, immunogenicity of a MAb is only a problem when the MAb is administered repeatedly. Although uncommon, adverse reactions like anaphylactic reactions can occur (Riethmüller et al, 1994). Furthermore, rapid clearance of infused MAb will occur upon subsequent administrations resulting in a diminished targeting efficiency of the MAb to the tumour (Khazaeli et al, 1994). In the present study, mU36 IgG appeared to be less immunogenic than E48 IgG. Up to now just three of 18 patients receiving 1–52 mg of mU36 IgG have shown a HAMA response when measured in the mU36 IgG-related HAMA assay, and this frequency may fall when cU36 IgG is used in RIT studies.

In this and previous papers, we have shown biodistribution data indicating that mE48 IgG and mU36 IgG are equally well suited for targeting antigen-positive tumour deposits. Because of its more homogeneous reactivity pattern, however, we think that MAb U36 is more generally applicable and therefore more suitable for RIT than MAb E48. Based on the aforementioned considerations, we will give priority to a phase I RIT trial with $^{186}$Re-labelled cU36 IgG in head and neck cancer patients with inoperable local or regional recurrences or distant metastases. In this radiation dose-finding study, patients will receive a single injection of the radioimmunoconjugate at a protein dose of 50 mg. When $^{186}$Re-labelled cU36 IgG is well tolerated and dosimetry estimations show the feasibility of RIT, an adjuvant RIT trial will be started.

### ACKNOWLEDGEMENTS

The authors thank Ing Corlinda BM ten Brink for immunohistochemistry, Dr Abraham J Wilhelm for pharmacokinetic determinations, Ing Henri Greuter for biopsy measurements, Ing Harry Twaalfhoven for HAMA determinations, Ronald Schoots and Dr Frank B van Gog for ADCC testing and Dr Ir Arthur van Lingen for dosimetry calculations. This work was supported by the Dutch Ministry of Economic Affairs and by Centocor Europe, Inc., Leiden, The Netherlands.

### REFERENCES

Blumenthal RD, Fand I, Sharkey RM, Boerman OC, Kashi R and Goldenberg D (1991) The effect of antibody protein dose on the uniformity of tumor distribution of radio-antibodies: an autoradiographic study. *Cancer Immunol Immunother* **33**: 351–358

Brakenhoff RH, Van Gog FB, Looney JE, Van Walsum M, Snow GB and Van Dongen GAMS (1995) Construction and characterization of the chimeric monoclonal antibody E48 for therapy of head and neck cancer. *Cancer Immunol Immunother* **40**: 191–200

Breitz HB, Weiden PL, Vanderheyden J-L, Appelbaum JW, Bjorn MJ, Fer MF, Wolf SB, Ratliff BA, Seiler CA, Foisie DC, Fisher DR, Schroff RW, Fritzberg AR and Abrams PG (1992) Clinical experience with rhenium-186-labeled monoclonal antibodies for radioimmunotherapy: results of phase I trials. *J Nucl Med* **33**: 1099–1112

Buist MR, Kenemans P, Van Kamp GJ and Haisma HJ (1995*a*) Minor human antibody response to mouse and chimeric monoclonal antibodies after a single i.v. infusion in ovarian carcinoma patients: comparison of five assays. *Cancer Immunol Immunother* **40**: 24–30

Buist MR, Kenemans P, Molthoff CFM, Roos JC, Den Hollander W, Brinkhuis M and Baak JPA (1995*b*) Tumor uptake of intravenously administered radiolabeled antibodies in ovarian cancer patients in relation to antigen expression and other tumor characteristics. *Int J Cancer* **64**: 92–98

Crippa F, Bolis G, Seregni N, Gavoni N, Scarfone G, Ferraris C, Buraggi GL and Bombardieri E (1995) Single-dose intraperitoneal radioimmunotherapy with the murine monoclonal antibody 1-131 MOv18: clinical results in patients with minimal residual disease of ovarian cancer. *Eur J Cancer* **31A**: 686–690

De Bree R, Roos JC, Quak JJ, Den Hollander W, Snow GB and Van Dongen GAMS (1994*a*) Clinical screening of monoclonal antibodies 323/A3, cSF-25, and K928 for suitability of targeting tumors in the upper-aerodigestive and respiratory tract. *Nucl Med Commun* **15**: 613–627

De Bree R, Roos JC, Quak JJ, Den Hollander W, Van Den Brekel MWM, Van Der Wal JE, Tobi H, Snow GB and Van Dongen GAMS (1994*b*) Clinical imaging of head and neck cancer with technetium-99m-labeled monoclonal antibody E48 IgG or $F(ab')_2$. *J Nucl Med* **35**: 775–783

De Bree R, Roos JC, Quak JJ, Den Hollander W, Wilhelm AJ, Van Lingen A, Snow GB and Van Dongen GAMS (1995*a*) Biodistribution of radiolabeled monoclonal antibody E48 IgG and $F(ab')_2$ in patients with head and neck cancer. *Clin Cancer Res* **1**: 277–286

De Bree R, Roos JC, Quak JJ, Den Hollander W, Snow GB and Van Dongen GAMS (1995*b*) Radioimmunoscintigraphy and biodistribution of monoclonal antibody U36 in patients with head and neck cancer. *Clin Cancer Res* **1**: 591–598

Gerretsen M, Visser GWM, Van Walsum M, Meijer CJLM, Snow GB and Van Dongen GAMS (1993) $^{186}$Re-labeled monoclonal antibody E48 immunoglobulin G-mediated therapy of human head and neck squamous cell carcinoma xenografts. *Cancer Res* **53**: 3524–3529

Gerretsen M, Visser GWM, Brakenhoff RH, Van Walsum M, Snow GB and Van Dongen GAMS (1994) Complete ablation of small squamous cell carcinoma xenografts with $^{186}$Re-labeled monoclonal antibody E48. *Cell Biophys* **24**: 135–142

Goldenberg DM (1989) Future role of radiolabeled monoclonal antibodies in oncological diagnosis and therapy. *Semin Nucl Med* **19**: 332–339

Hermanek P and Sobin LH (eds) (1987) *TNM Classification of Malignant Tumors*, 4th edn. pp. 13–26. Springer Verlag: Berlin

Jacobs AL, Fer M, Su FM, Breitz H, Thompson J, Goodgold H, Cain J, Heaps J and Weiden P (1993) A phase-I trial of a rhenium 186-labeled monoclonal antibody administered intraperitoneally in ovarian carcinoma: toxicity and clinical response. *Obstet Gynecol* **82**: 586–593

Khazaeli MB, Conry R and Lobuglio AF (1994) Human immune response to monoclonal antibodies. *J Immunother* **15**: 42–52

Oosterwijk E, Brander NH, Divgi CR, Welt S, Wakka JC, Finn RD, Carswell EA, Larson SM, Warnaar SO, Fleuren GJ, Oettgen HF and Old LJ (1993) Antibody localization in human renal cell carcinoma: a phase I study of monoclonal antibody G250. *J Clin Oncol* **11**: 738–750

Parker SL, Tong T, Bolden S and Wingo PA (1996) Cancer statistics, 1996. *C A Cancer J Clin* **46**: 5–27

Quak JJ, Van Dongen GAMS, Balm AJM, Brakkee JPG, Scheper RJ, Snow GB and Meijer CJLM (1990) A 22-kd surface antigen detected by monoclonal antibody E48 is exclusively expressed in stratified squamous and transitional epthelia. *Am J Pathol* **136**: 191–197

Riethmuller G, Schneider-Gadicke E, Schlimok G, Schmiegel W, Hoffken K, Gruber R, Pichlmaier H, Hirche H, Pichlmayr R, Buggisch P, Witte J and the German Cancer Aid 17-1A Study Group (1994) Randomised trial of monoclonal antibody for adjuvant therapy of resected Dukes'C colorectal carcinoma. *Lancet* **343**: 1177–1183

Schrijvers AHGJ, Quak JJ, Uyterlinde AM, Van Walsum M, Meijer CJLM, Snow GB and Van Dongen GAMS (1993) MAb U36, a novel monoclonal antibody successful in immunotargeting of squamous cell carcinoma of the head and neck. *Cancer Res* **53**: 4383–4390

Siegel JA, Wessels BW, Watson EE, Stabin MG, Vriesendorp HM, Bradley EW, Badger CC, Brill B, Kwok CS, Stickney DR, Eckerman KF, Fisher DR, Buchsbaum, DJ and Order SE (1990) Bone marrow dosimetry and toxicity for radioimmunotherapy. *Antibody Immunoconjug Radiopharm* **3**: 213–233

Stell PM and Rawson NSB (1990) Adjuvant chemotherapy in head and neck cancer. *Br J Cancer* **61**: 779–787

Steplewski Z, Sun LK, Shearman CW, Ghrayeb J, Daddona P and Koprowski H (1988) Biological activity of human-mouse IgG1, IgG2, IgG3, and IgG4 chimeric monoclonal antibodies with antitumor specificity. *Proc Natl Acad Sci USA* **85**: 4852–4856

Stupp R, Weichselbaum RR and Vokes EE (1994) Combined modality therapy of head and neck cancer. *Semin Oncol* **21**: 349–358

Takahashi H, Wilson B, Ozturk M, Motte P, Strauss W, Isselbacher KJ and Wands JR (1988) In vivo localization of human colon adenocarcinoma by monoclonal antibody binding to a highly expressed cell surface antigen. *Cancer Res* **48**: 6573–6579

Van Gog F, Visser GWM, Klok R, Van Der Schors R, Snow GB and Van Dongen GAMS (1996) Monoclonal antibodies labeled with rhenium-186 using the MAG3 chelate for clinical application: relationship between the number of chelated groups and biodistribution characteristics. *J Nucl Med* **37**: 352–362

Van Hal NLW, Van Dongen GAMS, Rood-Knippels EMC, Van Der Valk P, Snow GB and Brakenhoff RH (1996) Monoclonal antibody U36, a suitable candidate for clinical immunotherapy, recognizes a CD44 isoform. *Int J Cancer* **60**: 520–527

Vernham GA and Crowther JA (1994) Head and neck carcinoma – stage at presentation. *Clin Otolaryngol* **19**: 120–124

Watson EE and Stabin M (1984) Basic alternatives software package for internal radiation dose calculations. In *Computer Applications in Health Physics*,Kathren RL, Higby DP and McKinney MA (eds), *Proc 17th Midyear Topical Symposium of the Health Physics Society*, pp. 4.49–4.58. Columbia Chapter, HPS, Richland: Washington.

Weber DA, Eckerman KF, Dillman LT and Ryman JC (eds) (1989) MIRD: radionuclide data and decay schemes. The Society of Nuclear Medicine Inc: New York

Wessels BW, Harisiadis L and Carabell SC (1989) Dosimetry and radiobiological efficacy of clinical radioimmunotherapy. *J Nucl Med* **30**: 827

*© Cancer Research Campaign 1997*